# Impingement of suspending droplets with coming ones at different off-central distances: A molecular dynamics study

**DOI:** 10.1371/journal.pone.0330359

**Published:** 2025-09-12

**Authors:** Liwei Sun, Ye Han, Wei Shen, Chuiye Meng, Baocheng Zhan, Jiachao Gu

**Affiliations:** 1 School of Mechanical Engineering, Changchun Technical University of Automobile, Changchun, China; 2 FAW-Volkswagen Automotive Co., Ltd, Changchun, China; University at Buffalo, UNITED STATES OF AMERICA

## Abstract

Impingement of binary nanodroplets on solid surface can produce various outcomes, which are very important to various practical applications, such as lab on a chip and digital microfluidics. In the present work, we aim at exploring impacting systems with suspened nanodroplets and coming ones at various conditions. The effect of off-central distances, intrinsic contact angles, and Weber number is studied with the help of observing free evolution of targeted systems and several important characteristic parameters. This work reveals the effect of the off-central distance that is one of the most important characteristic parameters in impacting dynamics, mainly including generating rotating effect, spreading behavior with asynchronous dynamics, and hysteretic bouncing dynamics. The finding is very helpful for improving the understanding of off-central implementation of targeted systems, which can guide the practical application that needs to control outcomes of impacting binary nanodroplets.

## 1. Introduction

The impingement of water droplets upon solid surfaces is one of the most common phenomena innature and daily life. Recently, research has paid great attention to this topic because of its potential applications in inkjet printing [1], wing anti-ice [2], spray cooling [3], self-cleaning [4,5], and so on. Since the first work focused on impacting water droplets [6], numerous works following this pioneering research have investigated the impact process and devoted themselves to revealing physical mechanisms underlying it through theoretical analyses, experimental tests, and numerical simulations [7–10]. The representative dynamics have been well-drawn before. The impacting droplet initiates with spreading on solid surfaces, followed by retraction and deposition [11]. The energy analysis is widely adopted to explain the topologicalevolution over impingement of water droplets [12,13]. The process of spreading is a consequence of energy conservation between kinetic energy and surface energy. Therefore, the droplets’ surface energy continues to increase till the spreading droplet reaches its maximum value at the maximum extension state. The retraction is accompanied by released surface energy, and the retraction finally ceases to form the stable wetted droplet.

The phenomenon of impacting droplets results from a variety of competitive forces, typically including inertial, capillary, and viscous forces [14,15]. For example, the inertial force is found to promote deformation of impacting water droplets; in contrast, the viscous force plays an opposite role. Apart from the mentioned competitive forces, the physical property of working fluids (viscosity, density, and surface tension) also affects dynamic characteristics. To simplify the focused systems, several dimensionalparameters are developed toquantify these influencing factors. Two representative parameters of Weber number (*We*) and Reynolds number (*Re*) are introduced to simplify the issue, expressed as *We* = *ρD*_0_*V*_0_^2^/*γ* and *Re* = *ρD*_0_*V*_0_/*μ*, respectively. The *We* stands for the ratio of inertial force to capillary force, while the *Re* stands for the ratio of inertial force to viscous force. Where *ρ*, *γ*, and *μ* are density, surface tension, and viscosity, *V*_0_ is the given velocity of impacting droplets, and *D*_0_ is the diameter of droplets being in a spherical shape. Particularly, there are several important parameters for describing the dynamic evolution of impacting systems, including maximum spreading diameter, contact time, and restitution coefficient [16–20]. For example, the maximum spreading diameter could directly determine the precision of ink-jet printing or the absorption rate of pesticide spraying. The maximum spreading diameter is commonly nondimensionalized by the maximum spreading factor, expressed as *β*_max_ = *D*_max_/*D*_0_, where *D*_max_ is the diameter of the spreading droplet at its maximum extension state. Up till now, several scaling laws have been proposed to establish the relationship between *β*_max_ and *We* or *Re*. According to different dominant forces, two asymptotic regimes, referred to as capillary and viscous regimes, have been identified before [21–24]. In the viscous regime, the viscous force takes charge of impacting dynamics, and thus, the capillary force is out of consideration. Most of the input energy is dissipated by the so-called viscous dissipation, and the maximum spreading factor obeys a law of *β*_max_ ~ *Re*^1/5^ [25]. In contrast, the viscous force is so negligible that its effect can be omitted when the impingement falls into the capiilary regime. Many efforts have been devoted to exploring the universal law to predict the *β*_max_. Thus, the most famous law for describing spreading factor is *β*_max_ ~ *We*^1/4^, which shows a good agreement with experimental data over a wide range of hydrophicity and Weber number [24]. The contact time, *t*_c_, between impacting droplets and solid surfaces is also very important. It can reflect the bouncing kinetic characteristics, and the quick detachment of impacting droplets is very eager in use of anti-icing and self-cleaning. The contact time is defined as the time spent from initial contacting of droplets with solid surfaces to just leaving off surfaces. In general, the *t*_c_ is used in its dimensionless formation which is scaled as *τ*~(*ρD*_0_^3^/8*γ*)^1/2^ [25,26]. For the capiilary regime, the *τ* follows the inertia-capillary time with a value of about 2.3−4 [27]. The coefficient of restitution, *ε*_co_, can be used to quantitatively describe the efficiency of energy conversion during droplets’ impingement, defined as the ratio between bouncing kinetic energy to impacting kinetic energy. Hence, the value of the restitution coefficient is < 1. Aria and Gharib [28] further revealed the relationship between the restitution coefficient and the Weber number. They found that the coefficient of restitution followed the power law with regarded to Weber number. The coefficient obeys a law of *ε* ~ *We*^*-*1/4^ at low *We* range but changes to *ε*_co_ ~ *We*^*-*2/3^ as gradual increase in Weber number.

In recent years, the nanoscale impingement has become a vivid topic due to the rapid development of nanotechnologies, for example, nanoscale spray and nanoscale coating [29]. However, exploring impingement at the nanoscale is commonly difficult for conventional experimental test because of limitations in both time and space scales. Fortunately, molecular dynamics (MD) simulations can help researchers to achieve the purpose of investigating nanoscale dynamic behavior ranging from tens of angstroms to tens of nanometers [30,31]. The nanoscale impingement has several special features and scale effects, which lead to some novel phenomena. For the nanoscale impingement, the Ohnesorge number, *Oh* = *μ*/(*ρD*_0_γ)^1/2^, increases from *O* (10^−3^) to *O* (1), and hence, the viscous force becomes a non-negligible role even for low-viscosity fluid. Using MD simulations, Ma et al. [32] had drawn a phase diagram with respect to all the possible outcomes of impacting droplets upon solid surfaces. Besides traditional outcomes like deposit, bouncing, and breakup, the nanoscale impingement involves three new outcomes, referred to as holes rebound, partial-reboundsplash, and rebound splash. Ref. [33] discovered that the prominent viscous effect essentially comes from the velocity boundary being destroyed when the size of impacting droplets reduces to the nanoscale.

Although understanding of the nanoscale impingement has some important progress, impacting of binary droplets at the nanoscale is still very scarce. Actually, the multi-droplets’ impingement should be more close to practical applications, such as electronic packaging and rapid prototyping. The dynamic evolution of impacting binary macrodroplets had been captured in previous experimental tests [34,35]. Collisional droplets initiate with coalescence process that forms different coalescence patterns, mainly including temporary coalescence and permanent coalescence, depending on given impacting conditions [34]. It is well recognized that the temporary coalescence is only possible at large range of *We*. At such a situation, the opposite kinetic energy can overcome the cohesion between the temporary liquid bridge. As size of impacting droplets reduces to the nanoscale, the surface force significantly increases to prevent temporary coalescence from occurring [36]. Moreover, the collisional process of two suspended droplets with a off-centre distance adds the rotating behavior after a liquid bridge forms between collisional droplets [37]. However, up till now, the effect of solid surfaces on off-central impingement of two nanodroplets is incomprehensible, which is the most encountered situation for multi-droplets’ impingement. To fill this gap, we investigate the nanoscale impingement of a suspended droplet with a coming one via MD simulations. In the present work, the off-central distance, impacting Weber number, and intrinsic wettability of solid surfaces are three the most important parameters, and their effects have been explored systematacially. This can pave the way to understand the off-central impingement of two nanodroplets upon solid surfaces, and guide the practical applications with respect to impacting multiple nanodroplets.

## 2. Simulation method

**Fig 1:**
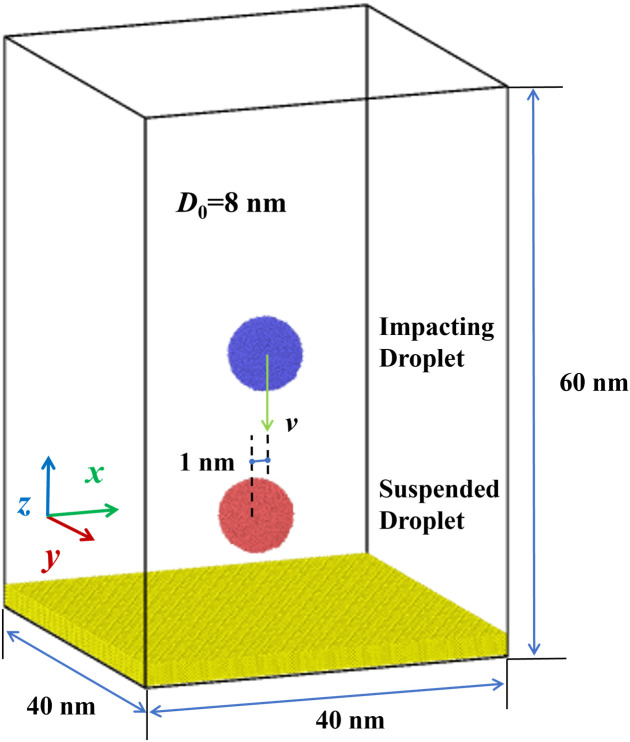
Simulated system in MD simulations, containing two nanodroplets with a diameter of 8 nm and a solid metal surface comprised of Pt atoms. Two nanodroplets are initially suspended in a vacuum environment with an off-centre distance of 1 nm.

This work uses molecular dynamics (MD) simulations performed with LAMMPS to simulate the impingement of binary nanodroplets with various off-centre distances on solid surfaces. The purpose of the work is to investigate the effect of characteristic parameters, including off-center distance, impacting Weber number, and surface wettability. Initially, we establish the configuration of MD simulations, as shown in Fig 1. Two water droplets are suspended in the vaccum, and the solid surface is consisted of Pt atoms. These two droplets are completely identical, and each of them has a diameter of *D*_0_ = 8 nm, containing 8900 water molecules. For initial configuration, the center-of-mass coordinates of two suspended droplets are (0, 0, 12) and (0, 0, 29), respectively. To explore the effect of off-centre distance (*D*_off_), the value of the *D*_off_ varies from 1 nm to 6.5 nm. In addition, we adapt an artificial virtual spring imposed on each metal atom to prevent the undesirable deformation of surfaces [32,38]. The simulated systems use periodic boundary conditions in each direction with dimensions of 40 nm, 40 nm, and 60 nm in *x*-, *y*-, and *z*- directions.

We use the mW water model to describe the intermolecular interactions among water molecules. The mW water model is a kind of coarse-grained model that only uses a larger group to simplify the structure of water molecules. Therefore, the computing cost is significantly reduced in comparison with the other models, such as TIP4P and SPC/E models [37–40]. Owing to the omission of the reorientation of the hydrogen atom, the viscosity of the model is *μ* = 283.7 μPa.s whose value is three times lower than the experimental value [29]. The intermolecular force for both water-Pt and Pt-Pt is described by the Lennard-Jones 12−6 potential, expressed as


$$U_{LJ}\left(r\right)=4\varepsilon \left[{\left(\frac{\sigma }{r}\right)}^{12}-{\left(\frac{\sigma }{r}\right)}^{6}\right],r<r_{cut}$$ (1)

where *r* is the distance separating two adjacent atoms, *ε* is the depth of the potential wall, *σ* is the zero-crossing distance, and *r*_cut_ is the cut off distance with a value of 1nm [35,37]. The interaction between water molecules is described by parameters of *ε*_w_ = 0.26838 eV and *σ*_w_ = 0.23925 nm, whose values change to *ε*_P_ = 0.69375 eV and *σ*_P_ = 0.247 nm to describe Pt-Pt interaction. Previous studies demonstrated that these parameters are effective in describing the dynamic behavior of nanoscale droplets, including impingement of nanoscale droplets, wetting transitions of nanoscale films, and spontaneous jumping of coalescence nanodroplets [38,39].

After that, we relax the system for 1 ns with a time step of 1 fs to obtain the equilibrium state. To achieve this purpose, the system is run in the NVT ensemble at 300 K over a pre-equilibrium process using the Nose–Hoover thermostat [29,40]. Subsequently, two droplets are run in the NVE ensemble for another 1 ns in productive processes. Impacting droplets are endowed with a series of vertical velocities to impact static ones in attempt to observe the dynamic evolution of targeted systems. The wettability of solid surfaces is generally controlled by a parameter of *ε*_w-Pt_, which can directly express the interaction between water and surfaces. Hence, we choose two values of *ε*_w-Pt_ as 0.0108 eV to 0.0045 eV to construct different intrinsic wettability from hydrophobic to superhydrophobic. To do so, we can investigate the effect of intrinsic wettability on the relevant dynamic behavior.

## 3. Results and discussion

**Fig 2:**
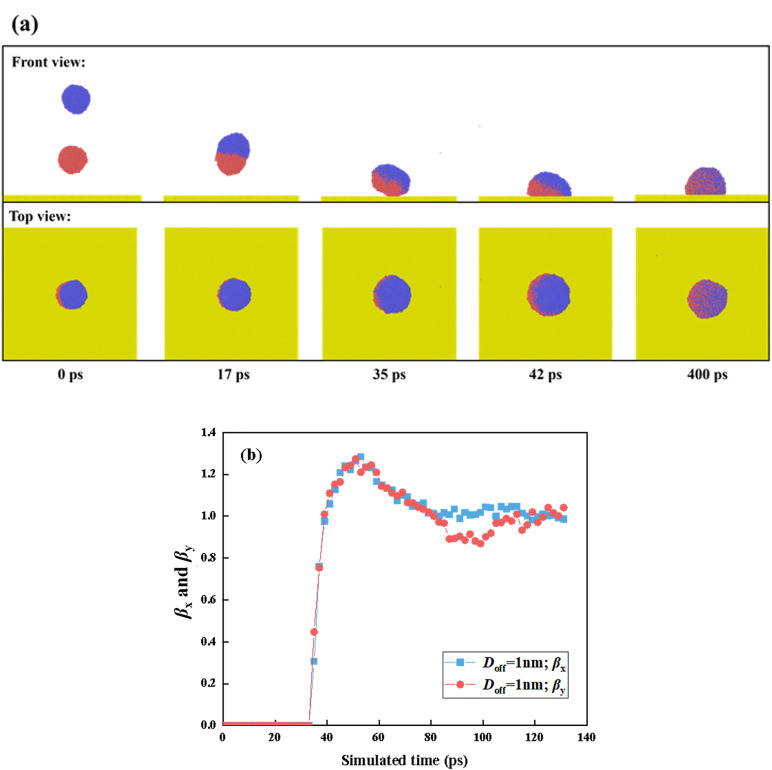
(a) Dynamic evolution of impacting binary nanodroplets with *D*_off_=11nm upon a solid surface with *θ*_Y_ = 102° at *We* = 18.15. (b) The corresponding spreading factor as a function of simulated time in both *x*- and *y*- directions.

**Fig 3:**
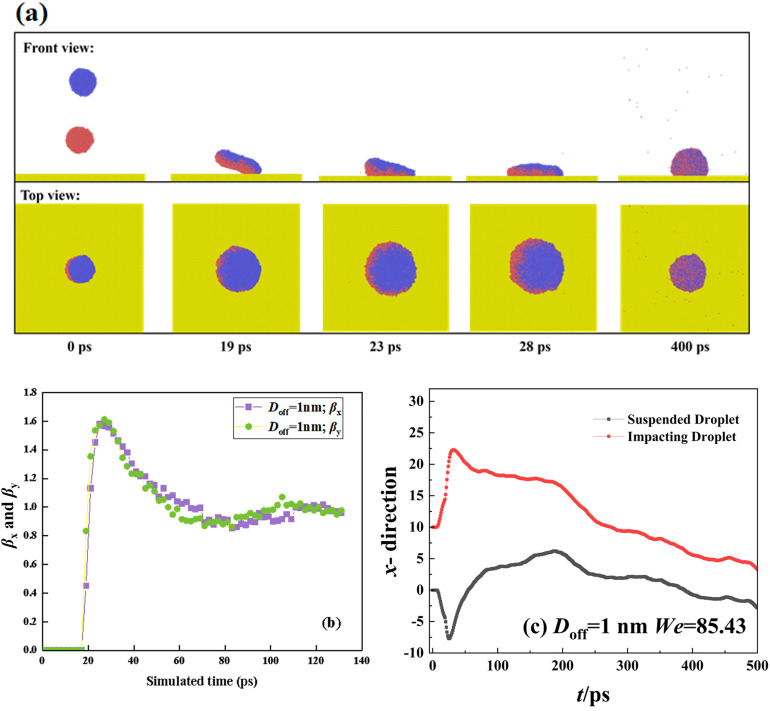
(a) Free evolution of targeted system with *D*_off_=1nm and *θ*_Y_ = 102° at *We* = 85.43. (b) Variation of spreading factor in both *x*- and *y*- directions. (c) Variation of mass center of two separated droplets along *x*- direction.

In this section, we observe the dynamic behavior of targeted systems at various Weber numbers in order to systematically investigate their effect, as shown in Figs 2 and 3. The off-central distance between droplets, *D*_off_, is initially fixed at 1 nm, and the intrinsic wettability is moderate hydrophobicity with *θ*_Y_ = 102° (*ε*_w-Pt_ = 0.0108 eV). At *We* = 18.15, snapshots show that the coalescence of separated droplet occurs with formation of a liquid bridge, and then the fast extension of the liquid bridge starts, forming a capsule shape with a deflected angle, as shown in Fig 2a. Since existence of *D*_off_ value, the impingement induces a very weak rotation of the merged droplet. The rotating effect of the merged droplet stems from the kinetic energy difference between the two original water droplets. As such, the rotation produces the asymmetric contact with the right side of the merged droplet preferentially touching the solid surface, see *t* = 35 ps. Next, we extract the evolution of the dimensionless spreading factor as a function of time after 35 ps, as shown in Fig 2b. For the specific systems containing impacting binary droplets, the dimensionless spreading factor is defined as the ratio of the wetted area of merged droplet’s spreading to its projection without any deformation. Although the asymmetric contact is observed, the spreading factor in both *x*- and *y*- directions increases synchronously, and thus, the procedure follows a very symmetric spreading feature. In addition, there is an obvious boundary between the original two droplets, showing a low degree of mutual diffusion. Restate, the progress of impacting droplets is a consequence of inertia, capillary, and viscous forces. The off-central distance has no influence on the spreading behavior because the small value of *D*_off_ induces a sufficient energy conversion between suspened droplet and coming droplet. The velocity within the merged droplet is relatively uniform, and thus, the capillary force plays an important role in controlling spreading behavior. The spreading droplet extends to the maximum extension state at *t* = 51 ps, and *β*_*x*_ and *β*_*y*_ reach a value of 1.24. Only when the merged droplet reaches its maximum extension can we observe the retracting behavior under the action of released surface energy. From Fig 2b, we observe that the retraction is also synchronous in each direction, apart from a short time interval (due to thermal disturbance). Ultimately, the droplet retracts to a stably wetted droplet with *β*_*x*_ = *β*_*y*_ ≈ 1, and a high degree of mutual diffusion with relatively uniform molecular distribution is attained, see *t* = 400 ps in Fig 2a.

**Fig 4:**
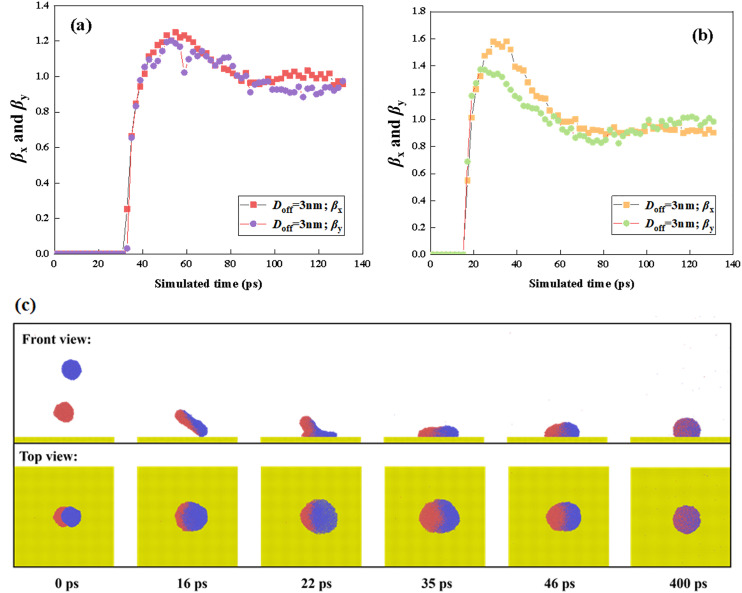
Variation of spreading factor at (a) *We* = 18.15 and (b) *We* = 85.43, showing asymmetrical spreading as Weber number increases. (c) Especially the dynamics of the targeted system at *We* = 85.43, which produces a liquid tail during the spreading stage.

**Fig 5:**
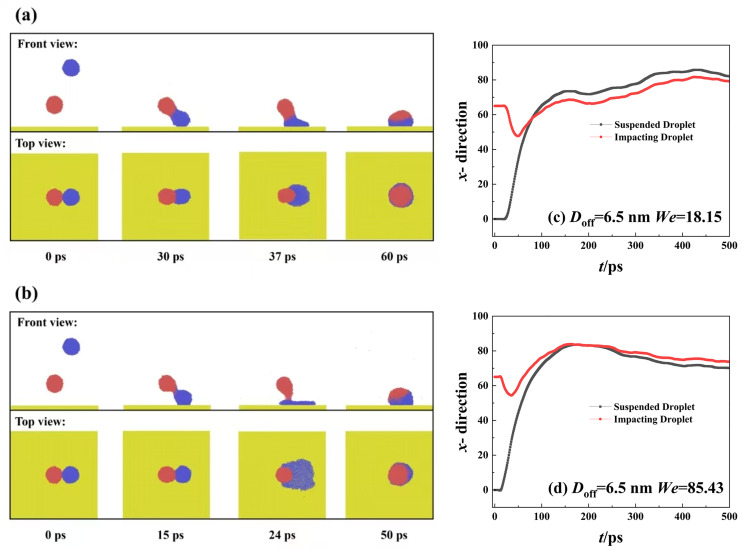
Two interesting phenomena of systems with *D*_off_=6.5 nm at (a) low Weber number of *We* = 18.15 and (b) high Weber number of *We* = 85.43.

**Fig 6:**
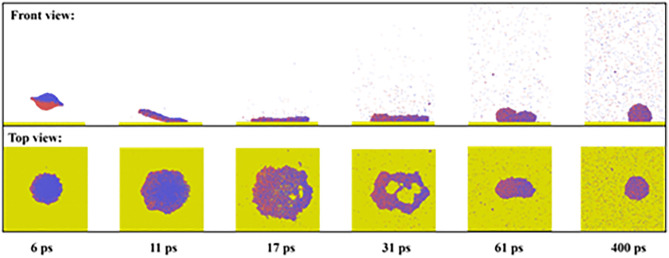
Snapshots of symmetrical spreading with the formation of the hole at the low off-central distance between two impacting nanodroplets at *We* = 277.25.

When the coming droplet is imposed on a high Weber number of *We* = 85.43, we observe a more violent process from coalescence to rotation, as shown in Fig 3a. However, the symmetric spreading can still not be broken down (see top view). This can be further confirmed by the evolution of the spreading factor in Fig 3b. Therefore, the capillary force can balance the asymmetric flow within the spreading droplet. We extract the variation of mass center of two droplets along the *x*- direction as a function of simulated time, as shown in Fig 3c. As increase in Weber number increases, the value of each mass-center coordinate increases but in the opposite direction (see Fig 3c). Hence, the merged droplet becomes a rotating liquid pancake before it spreads upon the surface, as shown at *t* = 19 ps. Soon after that, the spreading droplet forms a strange shape with the left side higher than the right side, see 23 ps in Figs 3a and 3c. Although the spreading in each direction is synchronous, the behavior of each droplets is actually different. The retraction of the original suspended droplet is faster than the other one due to forming peculiar shape in 23 ps. Subsequently, their rate gradually reaches the same value because the uniform distribution of water molecules. As the increase in *We*, the dimensionless spreading factor significantly increases to about 1.6 at the maximum spreading state, and a high retracting rate can also be observed from Fig 3b. However, the *β* finally returns to a value of 1, which is the same as that for impingement at *We* = 18.15. Therefore, the impacting *We* can not influence the final deposited droplet,which is a dynamic equilibrium between cohesion and capillary force. For off-central impingement upon solid surfaces, the off-central distance and intrinsic wettability of solid surfaces are the two most important parameters affecting impacting behavior. Thus, we mainly focus on the effect of these two mentioned parameters on targeted systems next. In the first step, we fix the intrinsic wettability at *θ*_Y_ = 102° to observethe variation of spreading factor with increasing *D*_off_ to *D*_off_=3 nm at impacting Weber numbers of 18.15 and 85.43, as shown in Figs 4a and 4b. At low *We*, the variation of the spreading factor is very similar to those observed in Fig 2a, indicative of inertial-capillary-dominant spreading. However, the different spreading behavior can be found as *We* increase to 85.43 (see Fig 4b), and the *β*_x_ and *β*_y_ no longer increase together after 22 ps. To obtain deep insights into this special behavior, we extract the snapshots from MD simulations to observe the corresponding dynamic evolution in detail, as shown in Fig 4c. The free evolution shows that the merged droplet initially rotates and forms a deflected water film before spreading, following a similar step as that for impingement with *D*_off_=1 nm. The increasing *D*_off_ increases the kinetic difference between the suspended droplet and the coming one, and thus, the progress of the merged droplet’s rotation enhancement, together with the increasing deflected angle. When the merged droplet reaches its maximum spreading in the *y*- direction, the spreading droplet forms a queer shape with leaving a small tail on the left side, see 22 ps in Fig 4c. The gradual flow from tail to bulk of spreading droplet makes the droplet further extend along the *x*-direction persistently, endowing spreading factor in the *x*-direction with a larger value, as shown at *t* = 35 ps (Figs 4b and 4c). Therefore, the asymmetrical feature is induced by the hysteretic flow of liquid-nail formation for the off-central impingement. From the top view of Fig 4c, we observe that the hysteretic flow allows the spreading droplet to take on a peanut shape over symmetrical spreading. During the retracting stage, the droplet retracts in the *x*- direction fastly than the other direction, expressing that the preferential spreading also promotes the retraction; and ultimately, the merged droplet forms a stable wettable droplet with *β*_*x*_ = *β*_*y*_ = 1. As the off-central distance increases to 6.5 nm, the hysteretic spreading of suspended droplets can be observed even at a low Weber number of 18.15, as shown in Fig 5a. The free evolution of the targeted system involves a dumbbell shape before the coming droplet starts to spread upon the solid surface, as shown at *t* = 30 ps. The original suspending and coming droplets are undergoing completely different evolution after 30 ps. The coming droplet first touches the surface with an increasing wettable area, leading to its mass center along *x*- direction incrases rapidly (see Fig. 5c). The dramatic spreading pulls the suspended one to flow into the spreading one, which results in a weak movement of the merged droplet toward the right side. The hysteretic spreading is more obvious when *We* increases to 85.43, the coalescing process between two collisional droplets forms a very slender liquid bridge, see Fig 5b This leads to a more violent increases in movement of mass center of coming one (Fig. 5d). Subsequently, the spreading behavior is quite adequate but the suspended droplet is almost immobile. The spreading and suspended ones pull each other through the liquid bridge so that the spreading behavior is also very asymmetrical. In addition, the spreading increases with the increasing Weber number, and the edge of the spreading droplet is not smooth and shows a distorted outline. This is due to the fact that the distance between adjacent water molecules significantly increases during spreading deformation, and the reduced van der Waals force can not maintain the smooth morphology of the spreading droplet. Therefore, the increase in the off-central distance can greatly increase the hysteretic spreading dynamics. Further increasing the Weber number to a very high value, we find that the slender liquid bridge can be destroyed over the preferential spreading droplet, forming two separated droplets again, and these two dropletscan finally coalesce into a merged droplet under the action of van der Waals force. These special dynamics are not mainly focused on in the present work, so we do not show the relevant snapshots.The asymmetrical feature is still found to be possible for the low value of the off-central-distance impingement but requires an extremely high Weber number, and the correspondingdynamic process has been recorded in Fig 6. As *We*increase to 277.25, there is a swelling that forms at the linking part between nanodroplets soon after droplets’contact, showing a suspended state in a saucer shape, see 6 ps. After that, the right part of the merged droplet starts to interact with the solid surface in a very thin water film. Owing to the significant increase in *We*, the kinetic energy difference is sufficiently high enough so that the asymmetrical dynamics with preferential extension in the *x*-direction occur 11 ps to 17 ps. At such a high Weber number, when the droplet reaches its maximum extension, the spreading droplet is unstable and forms several holes in the right part due to the thickness of the vibrational film reducing below a critical value. For the high-velocity impingement, the edge of the spreading droplet is not smooth and shows a zigzag outline, and the reason for this is also attributable to the weak van der Waals force among water molecules. Subsequently, the retraction occurs, and these tiny holes coalesce into a huge hole in the central part of the spreading droplet. From the top view of Fig 6, the retraction shows a particularretracting behavior with preferential recoil in the *y*- direction, opposite to the spreading behavior. Besides the asymmetric feature in both spreading and recoil stages, we also observe the directional motion of the retracting droplet from the left side to the right side. We find that the directional motion easily takes place at high *D*_off_ whereas the low *D*_off_ value requires much higher Weber number, and hence the directional motion is essentially due to the rotating effect induced by kinetic energy difference between original two nanodroplets.

**Fig 7:**
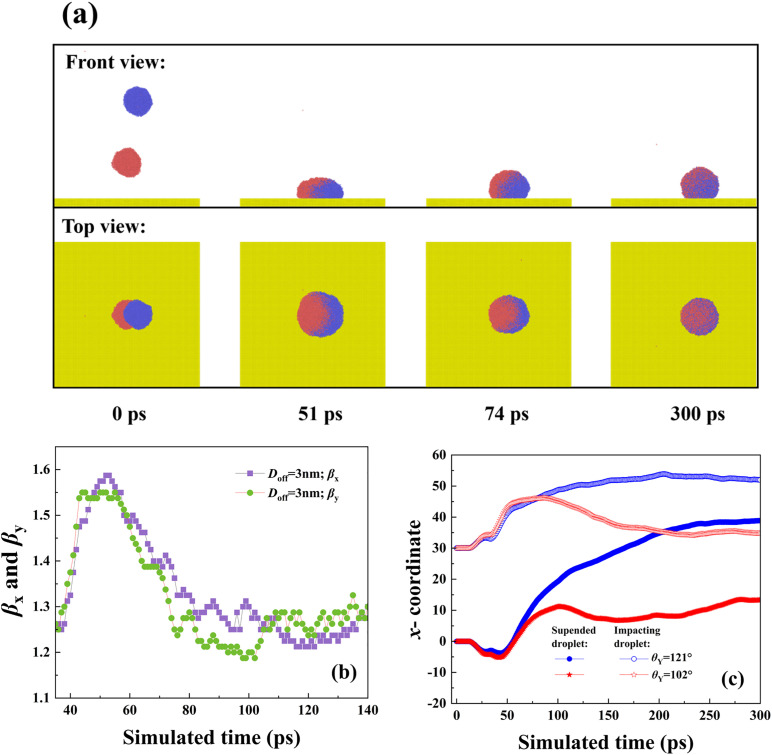
(a) Impacting nanodroplets upon anangled solid surface with increasing Young contact angle to *θ*_Y_ = 121°. (b) The variation of the spreading factor as a function of simulated time on the surface. (c) Varying the centroid of the nanodroplet over coalescence-spreading-retraction behavior.

**Fig 8:**
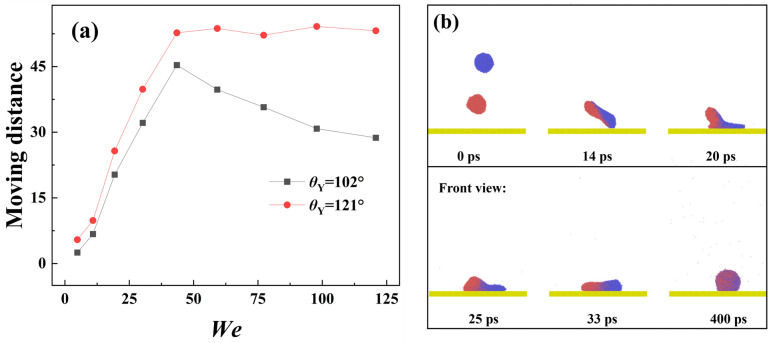
(a) Variation of moving distance for targeted systems with various contact angles as a function of Weber numbers. (b) The snapshots of abnormal variation of moving distance at *We* = 43.49.

**Fig 9:**
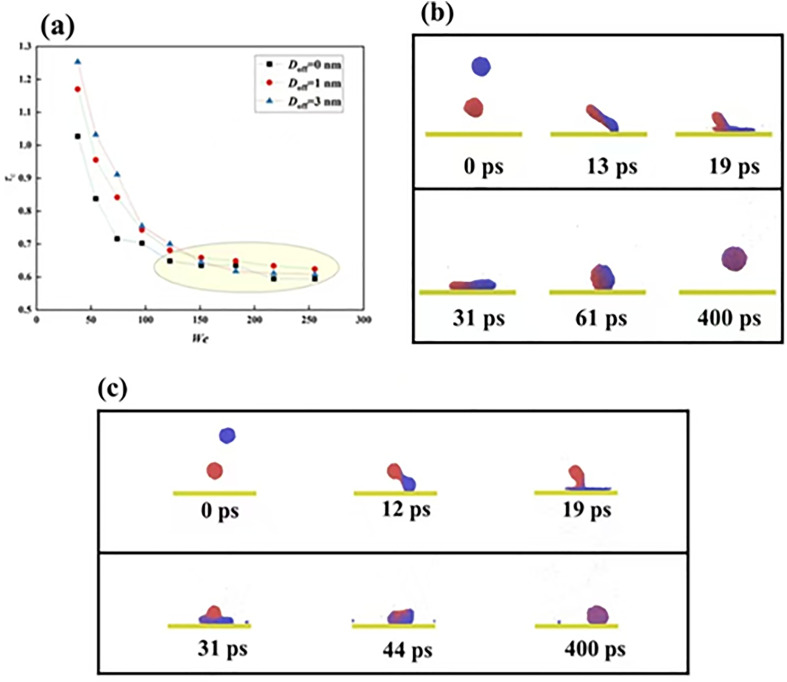
Variation of *τ*_c_ of targeted systems with *θ*_Y_ = 121° as a function of Weber numbers ranging from about 50 to 250. **(b)** Free evolution of an impingement-to-bouncing water droplet with *D*_off_=3 nm. **(c)** The free evolution of an impacting system of a suspended droplet with a coming one at *D*_off_=6.5 nm upon the superhydrophobic surface..

To explore the effect of intrinsic wettablity, we increase the Young contact angle from 102° to 121° to observe the free evolution of impacting binary nanodroplets, as shown in Fig 7a. The off-central distance in this section is still chosen as 3 nm, and the impacting Weber number is 18.15. The spreading in each direction shows exactly synchronous dynamics, indicating that the spreading behavior is not affected by varying *θ*_Y_. From this, we speculate that the spreading is also controlled by the inertia-capillary force. This has also been confirmed by the variation of spreading factor, as shown in Fig 7b, from which we observe that the two curves overlap with each other very well. The snapshots show that the merged droplet has an obvious tendency to move toward the right direction after the merged droplet achieves its maximum extension at about 50 ps. To obtain more detailed information with respect to directional motion, we record the variation of centroid for each nanoscale droplet of targeted systems with *D*_off_=3 nm upon solid surfaces with *θ*_Y_ = 102° and 121°, see Fig 7c. The centroid of the original two nanodroplets initially varies in the opposite direction; the coming droplet increases its centroid’s position toward the positive *x*-direction, but the suspended droplet shows the opposite motion. In addition, the centroid varies synchronously due to the inertia-forcepredominantprocess over merged droplets’ spreading. When the retracting stage starts, the stored surface energy returns to kinetic energy again, so that the predominant force changes. And, the kinetic energy induced by the recovery of the droplet’s deformation has a rotating effect to move the centroid’s position with increasing its value positively for the superhydrophobic case at *θ*_Y_ = 121°. However, for a surface with *θ*_Y_ = 102°, the intermolecular force is stronger,together with the capillary force. Therefore, there is only an unobvious phenomenon of centroid movement with its value initially increasing and subsequently decreasing. The increasing *θ*_Y_ is found to promote the movement of impacting binary nanodroplets. We next record the moving distance of the centroid at *θ*_Y_ = 102° and 121° at a wide range of *We*, as shown in Fig 8a. Herein, the moving distance respresents the variation of mass center of the merged droplet along the *x*- direction compared with the position with symcenter. From observation of the diagram, the movement distance at *θ*_Y_ = 121° is larger than that of *θ*_Y_ = 102°. In addition, the moving distance increases with the increase in the impacting Weber number for both *θ*_Y_ = 102° and *θ*_Y_ = 121° due to the enhancement of the rotating effect. For solid surface with *θ*_Y_ = 121°, the merged droplet can bounce off from the surface when the Weber number increases above 50, so the centroid is fixed at a constant value at *We* > 50. Intriguingly, we observe that, as progressive increase in impacting velocities, the Weber number changes from a favourable factor to anundesirable factor with the existence of a peak value for moving distance at *We* = 43.49. Further increasing the Weber number can lead the moving distance to reduce fastly. We show the corresponding dynamic evolution upon a solid surface with *θ*_Y_ = 102° at *We* = 59.19 to explain this abnormal phenomenon, as shown in Fig 8b. The snapshots show that the impacting water film first induces an obvious motion and attains its maximum spreading state, leaving a liquid tail generated by the suspended droplet, as shown at *t* = 20 ps. Af*t*er that, water molecules within the tail pattern gradually flow into the bulk droplet, leading to impair the behavior of directional motion. Hence, the behavior of fusion between the liquid tail and bulk liquid provides an extra energy to drag retracting droplet in the opposite direction against the initial movement. Finally, we focus on the bouncing behavior of targeted systems under different given conditions, including *D*_off_ and *We*. In addition, the bouncing phenomenon is one of the most important dynamics and is commonly characterized by contact time. Therefore, we recorded the variation of the contact time for impacting binary nanodroplets upon the surface with *θ*_Y_ = 121°, as shown in Fig 9a. Herein, the contact time is in a dimensionlessform and is expressed as *τ*_c_=(*ρμ*R**_0_^4^/*γ*_lv_^2^)^1/3^, and *R*_0_ is the radius of merged droplets and *γ*_lv_ is the liquid-vapor interfacial tension. The contact time is observed to decrease as *We* increases because more kinetic energy can be stored in the merged droplets’ deformation to promote the bouncing behavior. The snapshots show that the progress of merged droplet bounces after it undergoes spreading showing a nail shape and retracting, as shown in Fig 9b. The variation of contact time at *D*_off_=0 nm and 1 nm almost overlaps with each other because the free evolution of these two systems is very similar. Additionally, further increasing *D*_off_ is found to suppress bouncing formation associated with increasing contact time. This can be well explained by high-velocity-induced asymmetrical spreading, and liquid nail can consume extra energy. The contact time is invariant no matter how *We* increase and reaches a limited value at the same magnitude for all three systems. This is on account of impacting droplets acting as Hertzian spheres, as well described in Refs. 33. Therefore, it is reasonable to demonstrate that the progressive increase in *We* leads to a hysteretic feature to greatly increases *τ*_c,_ but the limited value is not changed. Further increasing *D*_off_, the bouncing dynamics can be completely suppressed when *D*_off_ increases to above 6 nm, and the corresponding free evolution is shown in Fig 9c. The hysteretic feature makes the original droplet spread fastly on the surface, and only formation of a fragile liquid bridge between the spreading droplet and the nail liquid. The flow of a nailed droplet into the original impacting droplet during its retracting stage, which consumes large amounts of energy, and the droplet ultimately deposits on the surface with a large directional movement at 400 ps.

## 4. Conclusions

In the present work, we mainly focus on effect of off-central distance on impacting dynamics of targeted systems containing a suspended droplet and a coming one via MD simulations. At a small *D*_off_, the effect of *D*_off_ can be safely neglected, with the spreading facor of merged droplets in each direction being synchronous. This is due to the fact that tha small off-central distance make the velocity within two droplets quickly uniform. On further increasing *D*_off_, the dynamic evolution of targeted systems is still very synchronous at the low *We* range. But the harmoniousscenario breaks down as *We* increases. There is a liquid nail which allows spreading factor along *x*- direction to further extend and forms an asymmetrical spreading. The increasing *D*_off_ is found to promote asymmetrical dynamics; in addition, the asymmetrical spreading is possible even for a low value of *D*_off_=1 nm at *We* = 277.25. The off-central impingement can induce rotating dynamics that can produce the direction of movement of merged droplets from left to right. The increasing *θ*_Y_ can promote the directional motion due to the weak intermolecular force between the liquid and solid. The directional movement occurs. Commonly, there is a peak value of moving distance because the liquid nail acts as an adverse factor. Finally, the effect of *D*_off_ on contact time is investigated and discussed. The contact time of targeted systems initially decreases and attains a constant value, which is analogous to that of impacting a single droplet. Before reaching limited contact time, the progressive increase in *D*_off_ plays a delayed role on contact time, with increasing its value at the same *We* because asymmetrical spreading needs to consume extra energy. The bouncing dynamics can be completely suppressed when the value of *D*_off_ increases above 6 nm. The nail liquid flows into the original impacting during the retracting stage, and the complete coalescence between the two separated droplets consumes all the energy provided by the spreading of the original impacting droplet.

### Nomenclature

**Table:** 

Abbreviations	
*We*	Weber number
*Re*	Reynolds number
*Oh*	Ohnesorge number
MD simulations	Molecular dynamics simulations
Symbols	
*ρ*	Density
*γ*	Surface tension
*μ*	Viscosity
*V*_0_	Velocity of impacting droplets
*D*_0_	Diameter of impacting droplets
*β*_max_	Maximum spreading factor
*t*_c_	Contact time
*τ*	Dimensionless contact time
*ε*_co_	Coefficient of restitution
*θ*_Y_	Young contact angle

## Supporting information

S1 Data Complete data file. See the supplementary material for all the data files related to the figures and the program files of molecular dynamics simulation.
(ZIP)
